# To explore the clinical efficacy of Traditional Chinese Medicine bath in the treatment of psoriasis vulgaris with blood-heat syndrome and its effect on related cytokines based on different temperature and different concentration

**DOI:** 10.1097/MD.0000000000020172

**Published:** 2020-05-08

**Authors:** Wenxia Lin, Qianying Yu, Yuesi Qin, Li Dai, Jianhua Xiao, Liang Jiao, Shan Liu, Shengzhen Ye, Jiahao Zhang, Mingling Chen

**Affiliations:** a Department of Dermatology, Hospital of Chengdu University of Traditional Chinese Medicine; b School of Clinical Medicine, Chengdu University of Traditional Chinese Medicine, Chengdu; c Chengdu Yinkang Psoriasis Hospital, Chengdu, Sichuan Province, PR China.

**Keywords:** Chinese herbal bath, herbal concentrations, psoriasis vulgaris, randomized controlled trials, water temperatures

## Abstract

**Background:**

Chinese herbal bath has long been used in the curative treatment of psoriasis vulgaris. However, there is no unified standard protocol for Chinese herbal bath. Many factors affect the curative effect of Chinese herbal bath, such as water temperature, bath concentration, and soaking time. Most studies involving Chinese herbal bath has described the bath generally, and few studies have investigated the factors that might contribute to the efficacy of Chinese herbal bath. Here we describe a protocol to evaluate the efficacy and safety of various bathwater temperatures and herbal concentrations on psoriasis vulgaris, and their effect on serum vascular endothelial growth factor (VEGF), tumor necrosis factor α (TNF-α), interleukin 23 (IL-23), and interleukin 17 (IL-17). These data could be useful for optimizing Chinese herbal bath treatments.

**Methods:**

In this randomized controlled trial, we planned to recruit 288 hospitalized atients with psoriasis vulgaris aged 18 to 65 years. All participants who meet the inclusion criteria will be randomly assigned to the observation group, the control group, or the basic treatment group. The observation group will be divided into 6 sub-groups according to water temperatures and bath concentrations, designated as observation groups 1 to 6. Thirty-six participants will be assigned to each group. The basic treatment group will be given co-qingdai capsule, po 2 g tid; compound glycyrrhizin tablet, po 75 mg tid; AA Skincare jojoba Oil, us.ext qd. The observation group will be given a Chinese herbal bath at the same time as the basic treatment. The control group will be given ozone hydrotherapy at the same time as the basic treatment. The entire treatment course will last for 2 weeks. The following parameters will be compared in each group, before and 2 weeks after treatment: the psoriasis area and severity index score (PASI), pruritus score, clinical efficacy, and dermatology life quality index score (DLQI); serum levels of serum VEGF, TNF-α, IL-23, and IL-17; and confocal laser scanning microscope images.

**Conclusion:**

This study will evaluate the efficacy and safety of various Chinese herbal bath conditions (water temperatures and herbal concentrations) on the treatment of psoriasis vulgaris, which will provide an important reference for the operation of Chinese herbal bath.

**Trial registration number:**

ChiCTR1900027468

## 1 Introduction

Psoriasis affects about 2% of the global population, and psoriasis vulgaris accounts for about to 90% of the incidence of psoriasis.^[1]^ Though the exact etiology and pathogenesis of psoriasis are not completely clear, there is a broad consensus that the immune system is involved in the pathogenesis of psoriasis. Through whole-genome analysis, most genes associated with psoriasis are immune-related. Psoriasis inflammation is mostly driven by innate and acquired immunity disorders.^[2]^ By promoting angiogenesis and the release of pro-inflammatory cytokines and chemokines, vascular endothelial growth factor (VEGF), tumor necrosis factor a (TNF-а), interleukin 23 (IL-23), and interleukin IL-17(IL-17) play important roles in the pathogenesis of psoriasis.^[3,4]^ Because of its complex pathogenesis, at present, psoriasis cannot be cured completely. Psoriasis treatments mostly aim to control and stabilize the disease, decrease the development process, reduce clinical symptoms (erythema, scale, plaque thickening, and pruritus), reduce short- and long-term adverse reactions, control psoriasis-related complications, and improve the patients’ quality of life.^[5]^

Traditional Chinese Medicine (TCM) has unique advantages in the treatment of psoriasis, and increasing incorporation of TCM into clinics has greatly improved psoriasis outcomes.^[6]^ However, there is as yet no unified standard protocol for Chinese herbal bath, and few studies have investigated the factors affecting the efficacy of Chinese herbal bath, such as water temperatures, bath concentrations, and soaking time.

To address this, here we plan to use Chinese herbal bath to treat psoriasis vulgaris patients, for testing multiple water temperatures and herbal concentrations. Based on the study outcomes, we will assess the severity of the psoriasis vulgaris and serum VEGF, TNF-α, IL-17, and IL-23 levels. These data will help to optimize Chinese herbal bath protocols and provide insights into the therapeutic mechanisms of Chinese herbal bath in the treatment of psoriasis.

## 2 Methods

### 2.1 Study design

**Figure 1:**
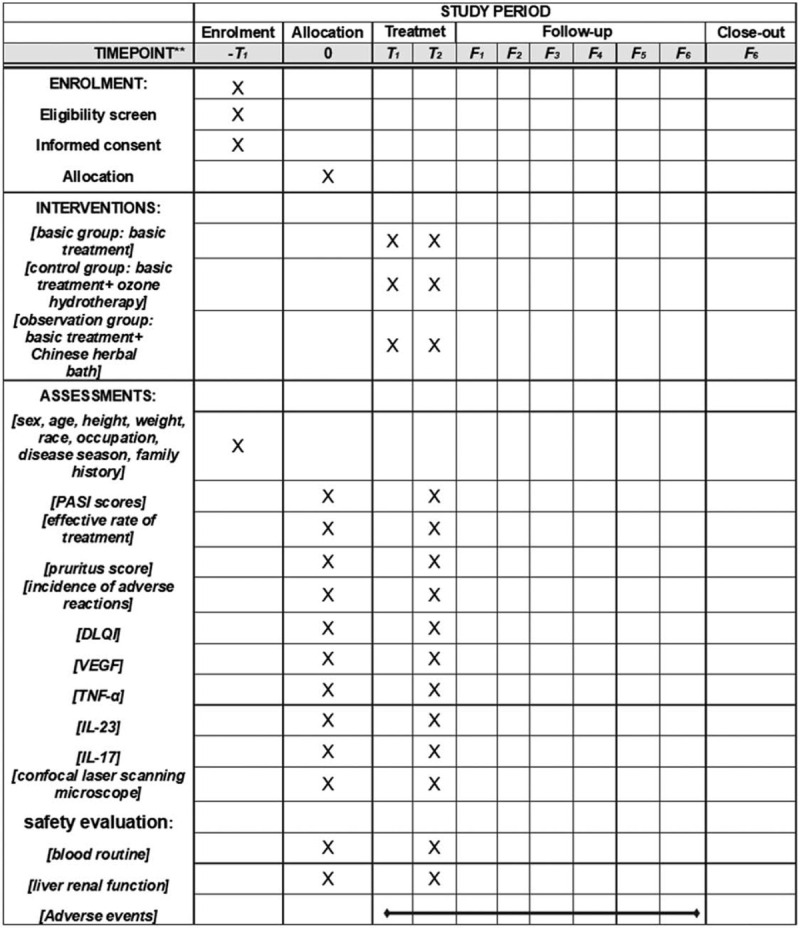
The schedule of enrolment, interventions, and assessments. DLQI = dermatology life quality index, F = months, IL-23 = interleukin 23, IL-17 = interleukin 17, PASI = Psoriasis area and severity index, T = weeks, TNF-α = tumor necrosis factor α, VEGF = vascular endothelial growth factor; .

**Figure 2:**
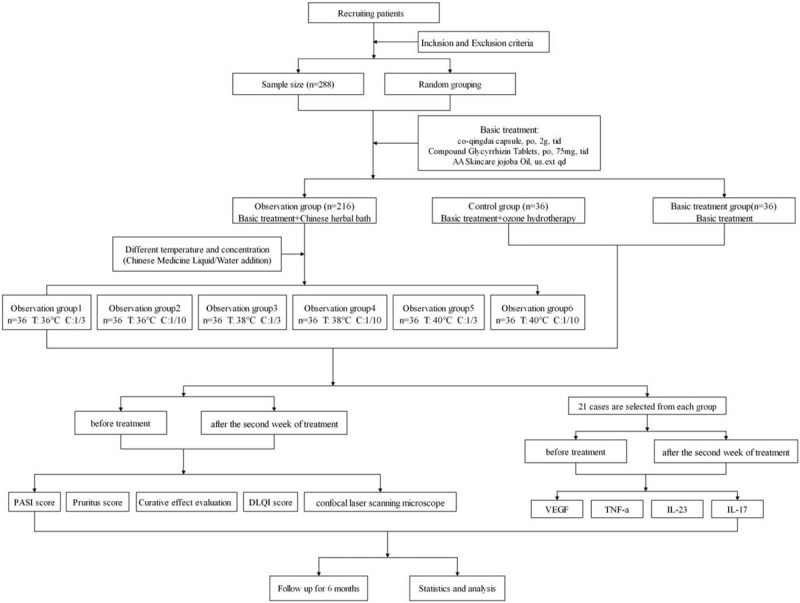
Flow diagram of study design.

The study has been planned as a single-center, parallel-group, randomized controlled trial (RCT). The Chinese Ethics Committee of Registering Clinical Trials has approved this study. Patients meeting the inclusion criteria will first be screened. All participants will sign informed consent forms, and they will be randomly assigned to the observation group, the control group, or the basic treatment group. Randomization will be performed through IBM SPSS Statistics 22.0 software's random number generator. Figure 1 presents the schedule of enrolment, interventions, and assessments. Figure 2 shows a flow chart of the study.

### 2.2 Participants

#### 2.2.1 Inclusion criteria

The inclusion criteria are: inpatients; patients corresponding to diagnosis standards psoriasis vulgaris with Western Medicine; patients corresponding to diagnosis standards psoriasis vulgaris with the blood-heat syndrome of Traditional Chinese Medicine; patients corresponding to the progressive stage of psoriasis vulgaris; patients diagnosed with psoriasis vulgaris between 18 and 65 years old, regardless of gender and course of the disease; patients that have provided informed consent, and voluntarily accept and cooperate with treatment, examination, and observation.

#### 2.2.2 Exclusion criteria

The exclusion criteria are: pregnant or lactating women; patients allergic to drugs or drug components; comorbid psoriatic arthritis, pustular psoriasis, or erythrodermic psoriasis; patients who have taken steroids within the preceding 2 weeks and/or taken retinoids or topical steroids within the preceding week; patients with severe primary diseases, such as cardiovascular, cerebrovascular, liver, kidney, or hematopoietic system diseases; patients with active pulmonary tuberculosis, acute or chronic hepatitis, or other infectious diseases; psychotic patients, epileptic patients, or other patients without self-restraint; external injury or infection on the body surface; and female patients with menstruation during hospitalization.

#### 2.2.3 Sample size calculation

According to the calculation formula of sample size in clinical trials,^[7]^ the necessary estimated loss rate is 10%, and the total sample size is 288 cases, with 36 cases per group. There will be 36 participants assigned to each group, including 36 cases in the basic treatment group, 36 cases in the control group, and 36 cases in each of the 6 observation groups.

#### 2.2.4 Randomization and blinding

Randomized grouping will be done using IBM SPSS statistics (version 22.0) software's random number generator.

#### 2.2.5 Allocation concealment

The random allocation form will be made in triplicate and kept in opaque envelopes. The project director, the pharmacy director, and the statistician will each keep one copy of the allocation form. At the end of the test, 3 random allocation forms will be simultaneously unsealed during a face-to-face meeting. If one or more of the seals are damaged, an explanation will be needed. Otherwise, the grouping information will be declared secret, or the test will be considered invalid.

#### 2.2.6 Blind method

Because the 3 groups’ intervention measures are not the same, blinding methods are not applicable.

#### 2.2.7 Participants recruitment

The participants will be recruited from the Hospital of Chengdu University of Traditional Chinese Medicine and Chengdu Yinkang Psoriasis Hospital by an open recruitment method. Participants will be screened according to the inclusion and exclusion criteria. The recruiting members will be Professor Chen Mingling, associate chief physician Dai Li, associate chief physician Xiao JIANHUA, attending physician Yu Qianying, and attending physician Qin Yuesi.

#### 2.2.8 Collection of general information of participants

The data will be collected by Liang Jiao and Shan Liu. The general information collected will include sex, age, height, weight, race, occupation, marital status, birthplace, home address, telephone number, disease season, past history, allergic history, and family history.

### 2.3 Intervention

#### 2.3.1 Basic treatment

All of the included cases will be given the basic treatment which has three components: a co-Qingdai capsule [recommended according to expert consensus on the treatment of psoriasis vulgaris with Chinese patent medicine (2014)^[8]^; manufactured by Tianning Pharmaceutical Co., Ltd., Shanxi Pharmaceutical Holding Group; National medicine permission number Z20010157; 0.5 g per granule; usage and dosage: po 2 g tid], glycyrrhizin tablets (recommended according to the guideline for the diagnosis and treatment of psoriasis in China, 2018 complete edition^[5]^; manufactured by Minophagen Pharmaceutical Co., Ltd; National medicine permission number J20130077; 25 mg per tablet; Usage and dosage: po 75 mg tid), AA Skincare jojoba oil (recommended according to the general topical therapies of psoriasis vulgaris^[9]^; manufactured by AA Skincare Ltd; National make-up with Chinese characters number J20128945; 100 ml per bottle; external use, once a day).

#### 2.3.2 The basic group treatment

The basic treatment group will be given only basic treatment.

#### 2.3.3 The control group treatment

The control group will be given ozone hydrotherapy at the same time as the basic treatment. The required tools are: a wooden bathtub, a disposable bathtub bag, cotton towels, slippers, and an ozone hydrotherapy instrument (Hunan Haizhi Medical Technology Co., Ltd., type HZ-261A).

The ozone water will be prepared as follows: the oxygen machine generates the oxygen that, via the ozone generator, generates a high ozone concentration (4 mg/L) in the bathwater. The participants will bath once per day, for 30 minutes.

#### 2.3.4 The observation group treatment

The observation group will be given Chinese herbal bath at the same time as the basic treatment.

The Chinese herbal bath prescription will be composed of: phellodendron (30 g), rhubarb (15 g), sophora flavescens (30 g), purslane (30 g), fructus kochiae scopariae (30 g), glycyrrhiza (30 g), mirabilite (20 g), alumen (10 g), and borneol (3 g).

All of the above Chinese herbs are provided by the Chinese medicine pharmacy of Chengdu Yinkang Psoriasis Hospital and decocted by the boiling machine of Chengdu Yinkang Psoriasis Hospital. According to the methods of the decoctum preparation and the standardization of decoction preparation in “Treatise on Exogenous Febrile Diseases,”^[10]^ and the management standard of Chinese medicine decoction room,^[11]^ the decoction amount is 1 L per dose. Table 1 shows Chinese medicine liquid and diluent of different concentrations.

**Table 1:**
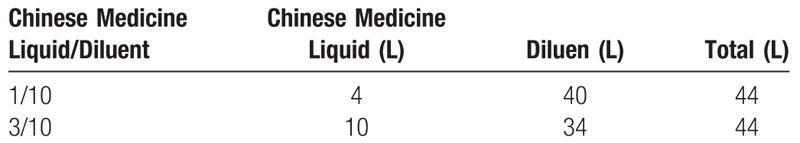
The Chinese Medicine liquid and diluent for different concentrations.

#### 2.3.5 The bathing process

For bathing, the following equipment is needed: a wooden bathtub, a disposable tub bag, cotton towels, slippers, and a constant temperature heater (Little Magneto Xiaoyubao, Manufacturer: Foshan Xiaociren Electric Appliance Industry Co., Ltd, model CA01).

Before the Chinese herb bath, the nurse will set the temperature of the bathroom to 20 to 22 °C,^[12]^ put the disposable tub bag in the bathtub, pour in the corresponding proportion of Chinese Medicine liquid and water, then add the constant temperature heater, setting the temperature and time. The patient will sit in the bath bucket, soak the torso and limb in the bathwater, and gently wipe their skin with a towel. The nurse will regularly check the situation in the bathroom and ask whether the patient has any discomfort, such as dizziness, dyspnea, palpitation, chest tightness, or other symptoms. If the patient reports any of the above discomforts, the herb bath will be stopped, and the nurse will assist the patients in returning to the ward, closely observe the condition, and ask the doctor to give treatment. After the Chinese herb bath is over, to avoid getting cold, the patients should thoroughly dry themselves with a towel. During the 2-week treatment period, the patient will take one herbal bath per day, with each bath lasting 30 minutes.

#### 2.3.6 Matters of attention when participating in Chinese herb bath

Women are forbidden from taking Chinese herb bath during menstruation. The participants should not fast while participating in the Chinese herb bath trial and avoid using bath products outside of the test.

#### 2.3.7 Trial course and follow-up

The treatment course will last 2 weeks, and the outcomes will be recorded before treatment and during the second week of treatment. After the 2 weeks of treatment, the patients will be followed up once per month for the next 6 months.

### 2.4 Primary outcome

Psoriasis area and severity index^[13]^ scores will be evaluated as the primary outcome. The PASI is a reliable, reproducible, and responsive instrument. This index has good internal consistency, intra-observer reliability, and inter-observer reliability, and is considered as the gold standard for assessing the severity of cutaneous manifestations in psoriasis. Therefore, this index will be used as our primary outcome.^[14]^

### 2.5 Secondary outcome

#### 2.5.1 The effective rate of treatment, pruritus score, and incidence of adverse reactions

The Guiding Principles for Clinical Research of New Traditional Chinese Medicine (trial) is the general evaluation standard in China. According to the evaluation standard, the effective rate of treatment, pruritus score, and incidence of adverse reactions are taken as secondary outcomes.^[15]^

#### 2.5.2 Dermatology life quality index

According to the DLQI questionnaire,^[16]^ patients need to complete ten questions about the impact of the disease on their quality of life (0 = none; 1 = slight; 2 = serious; 3 = very serious). The DLQI scores are calculated by summing the scores for each question. The maximum value is 30, and the minimum value is zero. The higher the scores, the greater the impact on the quality of life.

#### 2.5.3 Laboratory indicator

Using the IBM SPSS Statistics (version 22.0) random number generator, 22 patients will be selected from each group to detect serum VEGF, TNF-α, IL-23, and IL-17 levels before and after the second week of treatment.

#### 2.5.4 Confocal laser scanning microscope

Patients in each group underwent in confocal laser scanning microscope before and after the second week of treatment.

### 2.6 Safety evaluation

According to the safety assessment in randomized controlled clinical trials,^[17]^ the safety of this study will be evaluated by adverse events, routine blood tests, and liver and kidney function tests.

### 2.7 Data collection and management

All data will be analyzed according to the intention to treat principle. The missing value will be dealt with by carrying forward the most recent observation to the end point.

This study will use the IBM SPSS Statistics (version 22.0) software for data management and analysis. Professionally-trained dermatologists will collect all data. Members of the research team will perform the data collection and statistical analyses. Quantitative indicators will be expressed as mean ± standard deviation. If the sample is normally distributed and the variance is uniform, the *t* test will be performed: the paired sample *t* test will be used for intra-group comparison, and the independent sample *t* test will be used for inter-group comparison. If the variance is uneven, the approximate *t* test will be used. If the sample is not normally distributed, the rank-sum test (non-parametric) will be applied. The Chi-square test will be used for counting data. *P* values less than .05 will be considered as statistically significant.

The Chinese Ethics Committee of Registering Clinical Trials (Chengdu, China), which does not have any competing interests, will be responsible for monitoring the data. The Department of Science Research of the hospital at Chengdu University of TCM, which is independent of the investigators, will perform data audits in the trial.

## 3 Discussion

Psoriasis is a common chronic recurrent skin inflammatory disease. Psoriasis vulgaris plays a major role in the psoriasis. In the process of clinic, patients with psoriasis vulgaris are also the most common. The visiting time of patients is often in the progressive stage of psoriasis vulgaris, during this period, the original lesions gradually expand, or even fuse into pieces, new lesions continue to appear, which has the most serious impact on patients. With the gradual understanding of the pathogenesis of psoriasis and the continuous improvement of medical level, psoriasis has made great progress in treatment measures, but it is still in the stage of disease control and cannot be completely cured. Most patients need lifelong treatment. Long-term treatment, heavy economic burden, and easy recurrence have become the main factors affecting the quality of life of patients. Therefore, through the study of optimizing the treatment plan of psoriasis, reducing the economic burden of patients and delaying the time of recurrence, it is very important to formulate a reasonable treatment and follow-up plan, take targeted intervention measures and improve the quality of life of patients. The Chinese herb bath can achieve ideal curative effect by penetrating the drugs with warm effect into the skin or meridian points and directly into the meridians and blood vessels, acting on the focus site. After soaking in the bath of Chinese medicine liquid, the skin can remove pollutants such as exudates, crusts and scales, and has the effects of moisturizing and relieving itching, clearing heat and cooling blood, detoxification and convergence; Chinese medicine liquid can dilate the capillaries of patients and improve local microcirculation, promote metabolism, accelerate the repair of affected tissue, and promote the regression of skin lesions. In clinic, the use of Chinese herb bath, or Chinese herb bath combined with other therapies in the treatment of psoriasis is quite common, with high efficiency, definite curative effect, few adverse reactions and good patient compliance.

This study is an in-depth study on the Chinese herb bath in the treatment of psoriasis vulgaris. The research results can help the clinic optimize the treatment scheme of Chinese herb bath in the psoriasis vulgaris. Through the study of the Chinese herb bath in the treatment of psoriasis vulgaris and the relationship between VEGF, TNF- α, IL-23, IL-17 and psoriasis, to lay a theoretical foundation for clinical judgment of psoriasis vulgaris.

## 4 Contributors

Mingling Chen was the principal investigator of the study. Wenxia Lin and Qianying Yu contributed equally to this article, and both of them participated in the research design and the writing of the manuscript. Yuesi Qin, Li Dai, and Jianhua Xiao revised the original manuscript. Liang Jiao and Shan Liu involved in the recruitment of patients. Shengzhen Ye and Jiahao Zhang participated in data collection. All authors read and approved the final manuscript.

## Author contributions

**Conceptualization:** Wenxia Lin, Mingling Chen.

**Investigation:** Liang Jiao, Shan Liu.

**Supervision:** Li Dai, Jianhua Xiao.

**Writing – original draft:** Qianying Yu, Yuesi Qin.

**Writing – review & editing:** Shengzhen Ye, Jiahao Zhang.

Mingling Chen orcid: 0000-0002-8879-0857.
